# Hepatitis C in key populations in Latin America and the Caribbean: systematic review and meta-analysis

**DOI:** 10.1007/s00038-015-0708-5

**Published:** 2015-08-23

**Authors:** Monica Alonso, Annika Gutzman, Rafael Mazin, Carlos E. Pinzon, Ludovic Reveiz, Massimo Ghidinelli

**Affiliations:** Pan American Health Organization, HIV/STI/TB and Viral Hepatitis, 525 23rd St NW, Washington, DC 20037 USA; Pan American Health Organization, Knowledge Management, Bioethics and Research, Washington, DC USA

**Keywords:** Hepatitis C, Latin America, Caribbean, Drug users, Men who have sex with men, Prisoners

## Abstract

**Objectives:**

Summarize hepatitis C virus (HCV) prevalence in injecting (IDU) and non-injecting drug users (NIDU), men who have sex with men (MSM), sex workers, and prison inmates in Latin America and the Caribbean (LAC).

**Methods:**

Systematic review on HCV prevalence in sub-populations in LAC. Databases searched from 1-1-2000 to 10-30-2013. Inclusion criteria: prevalence studies in sub-populations in LAC. HCV-antibody was marker for prevalence of current/past HCV infection and HCV-RNA for prevalence of HCV current infection.

**Results:**

IDU HCV current/past infection presented highest prevalence, from 1.7 % in Colombia to over 95 % in Ciudad Juarez and Tijuana, Mexico and pooled regional anti-HCV prevalence was 49 % (CI 95 % 22.6–76.3 %). NIDU, MSM and sex workers anti-HCV prevalence was below 10 %, and pooled regional prevalence of 4 % (CI 95 % 2.6–4.5 %), 3 % (CI 95 % 1.7–4.5 %) and 2 % (CI 95 % 1.0–3.4 %), respectively. Prison inmates presented higher values, but prevalence decreased over the 15-year time span (*p* < 0.001). Current HCV infection from three countries showed prevalence under 10 % in prison inmates and 1–46 % among drug users.

**Conclusions:**

Disease burden is high and surveillance, prevention and treatment should target these groups in LAC.

**Electronic supplementary material:**

The online version of this article (doi:10.1007/s00038-015-0708-5) contains supplementary material, which is available to authorized users.

## Introduction

Hepatitis C virus (HCV) infection is a leading cause of chronic liver disease, cirrhosis and hepatocellular carcinoma (Perz et al. 2006). Hepatitis C causes chronic infection in almost 3 % of the world population and, since its discovery in 1989, has emerged as a worldwide public health concern (WHO 2010). Recent estimates for Central, South America and the Caribbean indicate HCV population prevalence levels between 1.5 and 3.5 % (Mohd Hanafiah et al. 2013). Furthermore, Brazil and Mexico together may have 4 million people with HCV (Szabo et al. 2012). The burden of HCV varies geographically and among subpopulations (Szabo et al. 2012; Kershenobich et al. 2011; Lavanchy 2011). Several studies from Latin America and the Caribbean (LAC) have reported a high prevalence among injecting drug users (Weissenbacher et al. 2003) and prison inmates (Guimaraes et al. 2001) as well as in other vulnerable populations, such as sex workers (Pando et al. 2006a, b), and men who have sex with men (MSM) (Pando et al. 2012). No systematic review has been conducted on HCV infection prevalence among specific population groups in LAC. To effectively respond to viral hepatitis C through provision of appropriate treatment and care services, it is critical to document a disproportionate impact and burden of disease among different population groups in LAC. This study aims to summarize available information on prevalence of hepatitis C infection in drug users, injecting and non-injecting, MSM, sex workers, male to female transgender populations and prison inmates in LAC.

## Methods

A systematic review and meta-analysis on hepatitis C prevalence in different population groups in LAC was conducted according to the PRISMA statement (Preferred Reporting Items for Systematic Reviews and Meta-Analyses) (Moher et al. 2009). Search terms (all fields) for the PubMed, LILACS and SciELO databases were: “hepatitis OR HAV OR HBV OR HCV OR HDV OR HEV” and “prevalence OR epidemiology” and, for the PubMed search only, a combination of the names of regions, countries and big cities in LAC (Electronic Supplemental Material, Table 1 presents the search terms used for the review). Searches included original articles in English, Spanish, French or Portuguese. We also screened the references of retrieved articles. One reviewer screened all abstracts and full texts for the inclusion criteria and conducted the data extraction. A second expert conducted data extraction on a random sample of studies.

The inclusion criteria consisted of the following: primary sources published between 01/01/2000 and 10/01/2013 with data on Hepatitis C prevalence in one or more of the following populations from LAC countries: Sex workers, MSM, transgender populations, prison inmates and drug users. We defined key populations as both most-at-risk populations and vulnerable populations according to the 2013 WHO classification for HIV (WHO 2013a, b). If several publications were based on the same study, only one of the publications was included. Exclusion criteria were studies with a sample size <75 and studies that did not mention the testing markers. The sample size cutoff criteria (75 participants or greater) were decided given the paucity of studies in the region.

**Fig. 1:**
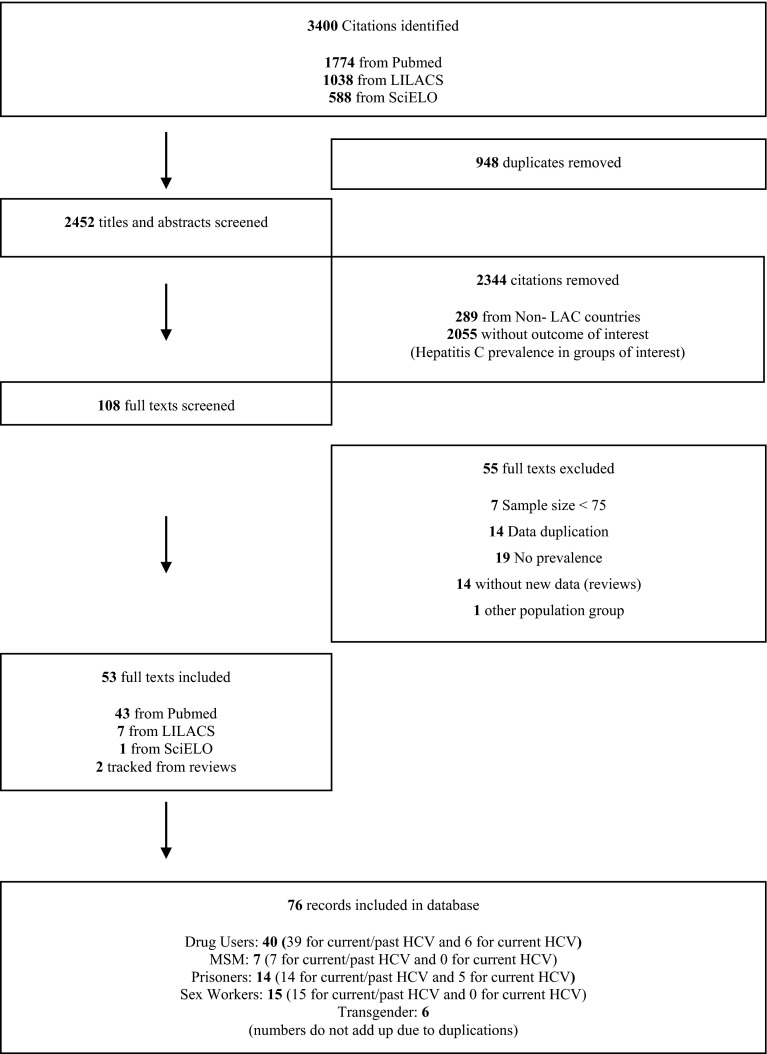
Flow diagram of the study selection procedure for the review on prevalence of current and/or past Hepatitis C infection in Latin America and the Caribbean from 2000 to 2013

The flow chart of the systematic review and meta-analysis is shown in Fig. 1.

Positivity to anti-HCV antibody was interpreted as current and/or past infection (current/past). HCV-RNA positivity was categorized as current infection. For HCV-RNA, prevalence was given as percentage of the whole study sample although testing was sometimes only done among anti-HCV positive individuals. Whenever possible, prevalence from confirmatory tests was used. Wilson confidence intervals were calculated for each prevalence point.

The quality of the included studies in the review was evaluated with the QATSO scale developed by Wong et col. (Wong et al. 2008). Two additional criteria were evaluated: the acceptability of the case definition for each subpopulation and for the outcome variable and the adequacy of the sample size.

### Data extraction

HCV prevalence was extracted from the studies based on hepatitis C marker (antibody or RNA) and by population group. Multiple center studies were split up into sites/cities when possible. Therefore, one reference could yield multiple records. Additional characteristics collected were gender, age group, genotype, data collection period, study setting, country and city or area, sample size, and type of laboratory test.

### Statistical analysis

Linear and multiple regression analysis evaluated the influence of year of field work on prevalence, using R statistical language and environment (R Core Team 2011). The analysis was applied within population groups if at least 5 data points were available. For country, dummy variables were created. The country having (1) the lowest prevalence and (2) more than one data point was selected as reference.

Meta-analysis was performed using STATA 12 Software Version 12.0. Prevalence was reported by 95 % confidence intervals (CIs). Random effects models were used, taking into account the possibility of heterogeneity between studies, which was tested with the Cochrane *Q* test and I2 test. We calculated prediction intervals (PI) to evaluate the dispersion of the estimated prevalence’s.

Subgroup analyses were conducted for results controlling (either by standardization or statistical adjustments) for risk group of infection and country. We performed analyses stratified by geographic area and year of field work.

## Results

A total of 3400 references were identified in the three databases (Fig. 1). Most of them were excluded after revising the abstracts. Fifty-three full text citations were included. Only seven studies from the LILACS and one from the SciELO database were included as most of the citations were already found in PubMed. Fifty five studies were excluded; seven due to small sample size, five among drug users, one among prison inmates and one among MSM, from Brazil, Puerto Rico and Mexico. Prevalence was in range with included studies.

Seventy-five data points from the following countries were available for current/past HCV infection: Argentina, Brazil, Colombia, Dominican Republic, Mexico, Panama, Peru, Puerto Rico, Uruguay and Venezuela. For current HCV infection, based on HCV-RNA, 11 data points from the following countries were included: Brazil, Mexico, and Venezuela. The baseline characteristics of the studies appear in Electronic Supplemental Material, Table 2.

All data for transgender populations were from studies focusing on sex workers or conflating transgender and MSM. Therefore, the transgender population was not investigated separately.

### Quality of studies

Results of the study quality assessment are provided in Electronic Supplemental Material, Table 3. Eighty-eight percent of studies reported sampling methods, most of them using non-probability sampling (70 %). Measurement of HCV was by laboratory testing for all studies. Thirteen studies had samples sizes of 100 or less and the median sample size of those with sample size >100 was 270 (interquartile range *Q*25–*Q*75: 197–500). All the studies used serological markers to diagnose hepatitis C infection and the testing assays were specified in 96 % of the studies. In the majority of cases, a single enzyme immunoassay was used. Confirmatory or supplementary testing was done by immunoblot assays in 20 % of records.

### Sex workers

**Fig. 2:**
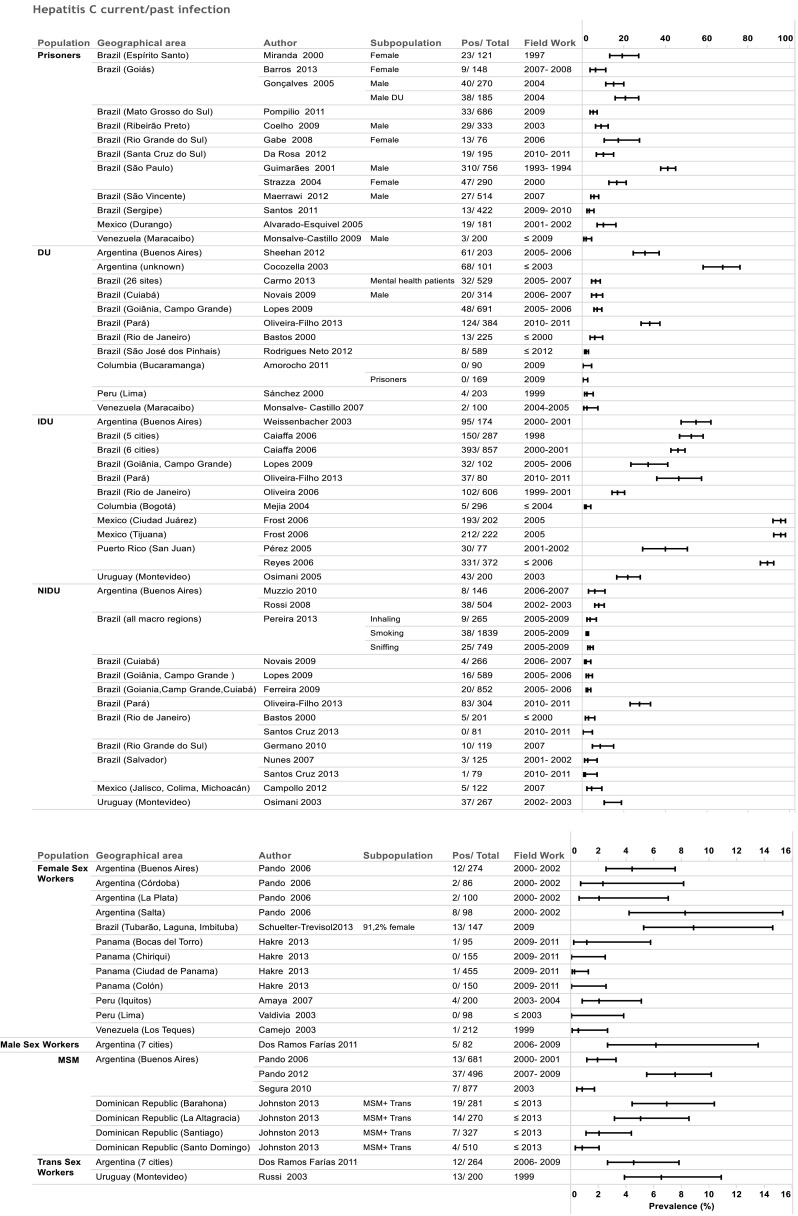
Forest plot for hepatitis C infection prevalence in Latin America and the Caribbean from 2000 to 2013. *DU* drug users, *IDU* injecting drug users, *NI DU* non-injecting drug users, *MSM* men who have sex with men

Eight studies met the inclusion criteria, leading to 15 data points on past/current HCV prevalence (Fig. 2) and no data on current HCV infection prevalence.

Most of the studies focused on female sex workers in five countries. The minimum age of participants was 18 years old, with average age around 27–29 years old in 8 studies, and three studies had average age of 33–36 years old. Current/past infection rate of HCV was below 2 % in Panama (Hakre et al. 2013), Peru (Valdivia et al. 2003; Guerra Amaya et al. 2007) and Venezuela (Camejo and Díaz 2003), and was 0 % in three locations (Chiriquí and Colón in Panama, and Lima, Peru). Highest values were in Argentina (Salta) and Brazil (Southern region of Santa Catarina State) with values up to 9 % (the latter study included 9 % male sex workers and 56 % of the sample inhaled illicit drugs). The pooled weighted estimate for current/past HCV infection among sex workers in Latin America based on available studies was 2 % (CI 95 % 1.0–3.4 %) with significant heterogeneity (*p* = 0.00) PI (0.56, 5.32).

Among transgender sex workers, current/past HCV prevalence was available from two studies with figures similar to those from studies among female sex workers in Argentina and counting among the higher results found in the region (7 % in Montevideo, and 5 % in seven cities in Argentina) (Dos Ramos Farias et al. 2011; Russi et al. 2003). The latter study also provided HCV prevalence for male sex workers (current/past HCV prevalence of 6 %).

### Men who have sex with men

Seven data points from Argentina and Dominican Republic were analyzed (Fig. 2). Two studies reported an average participant age of 30 years old and in four study sites the majority of participants (54–68 %) were <24 years old (Johnston et al. 2013). No studies used HCV-RNA assays, thus no data were available for current HCV infection. Three studies from Buenos Aires seem to support an increase in HCV prevalence between 2001–2003 and 2009 (Pando et al. 2006a, b, 2012; Segura et al. 2010). The pooled estimate value for current/past HCV prevalence was 3 % (CI 95 % 1.7–4.5 %) with statistically significant heterogeneity, PI (−1.12, 8.23).

### Prison inmates

Thirteen studies from Brazil, Venezuela (Monsalve-Castillo et al. 2009) and Mexico (Alvarado-Esquivel et al. 2005) provided 14 data points for analysis; the majority of studies were from Brazil (Fig. 2).

Multiple regression analysis indicated that anti-HCV prevalence in prisons has gone down within 15 years (*p* value 0.001). In Brazil, the oldest study from Sao Paulo with 18.6 % of inmates reporting previous intravenous drug use had an extremely high prevalence of 41 % (Guimaraes et al. 2001). The most recent study was from 2011 (Santa Cruz do Sul) and gave a prevalence of 10 % for anti-HCV (Rosa et al. 2012). In the 2004 study in a prison in Goia, HCV prevalence was 15 %, but increased to 21 % among drug users (Gonçalves 2005). Pooled prevalence for current/past HCV among inmate population was 12.5 % (CI 95 % 7.7–17.3 %) but heterogeneity was statistically significant (*p* < 0.000), PI (10.67, 19.51).

HCV-RNA indicating current HCV infection was analyzed in all three countries. In Brazil, prevalence was stable at around 3 % in three different studies (Santos et al. 2011; Barros et al. 2013; Pompilio et al. 2011). Prevalence in Venezuela (Monsalve-Castillo et al. 2009) was significantly lower than in Brazil. All except one study evaluated genotypes. Taken these studies together, genotypes were determined in 50 individuals, of which 90 % had genotype 1, being 1a the most common. 8 % had genotype 3.

In three of the five studies, more than 80 % of the anti-HCV positive cases were also HCV-RNA positive. Nevertheless, two studies from Brazil presented a much higher percentage of past infection (Barros et al. 2013; Pompilio et al. 2011).

No significant difference in HCV prevalence was seen by sex in the multiple regression analysis.

### Drug users

Eight countries presented data on hepatitis C prevalence in drug users (DU) resulting in 39 data points (Fig. 2), some distinguishing between injecting (IDU) and non-injecting drug users (NIDU) (Ferreira et al. 2009; Bastos et al. 2000; Campollo et al. 2012; Germano et al. 2010; Lopes et al. 2009; Muzzio 2010; Novais et al. 2009; Nunes et al. 2007; Osimani 2003; Pereira et al. 2013; Rossi et al. 2008; Santos Cruz et al. 2013).

Prevalence for anti-HCV in DU remained below 7 % in the majority of studies. However, infection rates for current/past HCV infection among DU were up to 67 % in Argentina in a study conducted in 2003 (Cocozella et al. 2003), and 30 % in 2006 (Sheehan et al. 2012) (prevalence of ever-having injected drugs in both samples was 43 %). A 2011 study from Brazil also showed a current/past HCV infection rate of 32 % (Oliveira-Filho et al. 2013). In DU among mental health patients, no difference in prevalence was seen for DU compared to other studies (Carmo et al. 2013).

Data for anti-HCV among NIDU ranged from 0 to 10 % with the highest values found in a recent study from Brazil (8 %) (Germano et al. 2010), and Uruguay (10 % in 2003) (Osimani and Latorre 2003). The pooled value was 3.6 % (CI 95 % 2.6–4.5 %), PI (2.34, 7.58).

HCV infection rate for IDU varied considerably between and within countries (Brazil, Puerto Rico). The highest values were found in Argentina (55 % in 2001) (Weissenbacher et al. 2003), Brazil (53 % in 1998, 46 % in 2001) (Caiaffa et al. 2006), Puerto Rico (89 % in 2006) (Reyes et al. 2006) and Mexico (Ciudad Juarez and Tijuana) (96 % in 2005) (Frost et al. 2006). A study from Colombia (Bogota) found the lowest anti-HCV prevalence (1.7 %) in IDU (Mejia and Perez 2004). The latter is in agreement with another study conducted in another part of Columbia that indicated an absence of anti-HCV in DU and also in prison inmates who were drug users (Bautista Amorocho et al. 2011). Pooled regional anti-HCV prevalence among IDU was 49 % (CI 95 % 22.6–76.3 %) with significant heterogeneity among studies (*p* < 0.01), PI (33.45, 62.76).

**Fig. 3:**
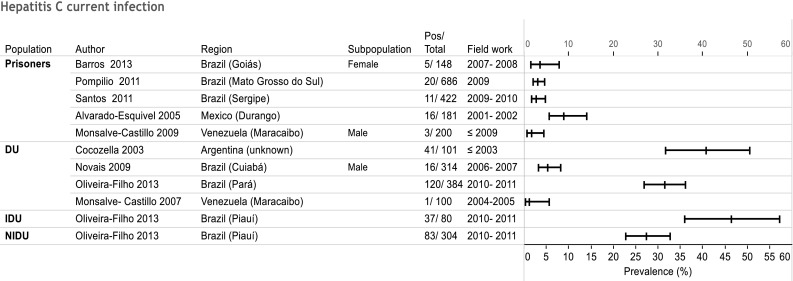
Forest plot for current Hepatitis C infection prevalence Hepatitis C infection in Latin America and the Caribbean from 2000 to 2013. *DU* drug users, *IDU* injecting drug users, *NIDU* non-injecting drug users

Four studies investigated both anti-HCV and HCV-RNA infection rate in DU, with six data points in total (Cocozella et al. 2003; Novais et al. 2009; Oliveira-Filho et al. 2013; Monsalve-Castillo et al. 2007) (Fig. 3). In all but one study (Oliveira-Filho et al. 2013), prevalence was much lower for HCV-RNA. HCV-RNA prevalence was much higher in IDU than in DU and NIDU in the study by Oliviera-Filho et al. who investigated all three populations. Only three studies from Brazil (Lopes et al. 2009; Novais et al. 2009; Oliveira-Filho et al. 2013) determined HCV genotypes. The study from Pará found a high prevalence of genotype 1b (42 %), especially in NIDU (50 %), while in the other two studies, individuals had genotype 1a in over 60 %.

### Comparison of results among population groups

For HCV current/past infection, IDU presented the highest prevalence (Fig. 2). The study from Mejia and Perez was the only to report a prevalence below 10 %. Among DU, NIDU belonged to the less at risk populations, similar to those found among MSM (with values below 10 %, range 0–10 %). SW, MSM and NIDU summary regional estimates for HCV current or past infection are 2, 3 and 3.6 %, respectively. Prison inmates presented higher values, with a regional value of 12.5 %, with prevalence rates declining over the years.

Information on HCV current infection prevalence was only available in two of the five groups of interest (Fig. 3). In general, it was lower than anti-HCV infection rates, ranging from 1 to 46 %. It was below 10 % in all studies on prison inmates.

## Discussion

To the best of our knowledge, this is the first review that systematically summarizes data on hepatitis C prevalence in five key populations from LAC. More than 50 % of studies investigated drug users. There was a significant lack of studies for male sex workers and the transgender population.

Nearly 50 % of all publications included were from Brazil. There are no data for many countries in the region, and studies from only ten countries were included overall. Whereby, the need for greater investment in HCV surveillance studies is high. As reported by the World Health Organization’s Global policy report on the prevention and control of viral hepatitis (WHO 2013a, b), more than 70 % of WHO member countries in LAC indicated that WHO assistance would be necessary for “viral hepatitis surveillance” and “estimating the national burden of viral hepatitis”.

As shown by this review, HCV prevalence results agree with the common epidemiological knowledge on HCV transmission routes. When analyzing HCV prevalence among MSM in the region (pooled 3 % anti-HCV prevalence), we must consider that there can be overlapping injecting drug use and HIV co-infection. Prevalence values appear in consonance with Buffington et al. who report 1.5–3.6 % HCV prevalence among non-injecting and injecting MSM, respectively, in several USA cities (Buffington et al. 2007) or 2.1 % HCV prevalence among MSM clinic attendees in England (Ward and Lee 2014). Results suggest that this population presents a higher risk of HCV than the general population highlighting the need for a greater focus on surveillance and services for MSM (Tohme and Holmberg 2010; Ponde 2011). The rate of infection due to sexual transmission is believed to be low, but factors such as unprotected sex with multiple sex partners, traumatic sex, co-infection with HIV or other STIs increase the risk of transmission and chronicity once infected with HCV (Terrault et al. 2013).

This review focused on vulnerable and risk groups that can have overlapping risk behaviors for HCV transmission. The authors recognize the limitations as information on overlapping risk behaviors in most cases was not readily available for this review.

This study updates and adds-on to reviews on HCV among injecting drug users worldwide (Aceijas and Rhodes 2007; Nelson et al. 2011). Our findings confirm Aceijas and Rhodes review that indicated important variation of HCV prevalence among IDUs in the region. The findings are also consistent with global figures. Nelson et al. found, globally, HCV prevalence among IDUS between 60 and 80 % in 26 countries, and in USA, Russia and China, countries with the largest amount of IDUs, HCV prevalence is estimated from 67 to 73 %. HCV prevalence among FSW was among the lowest found in some countries, and varying prevalence most likely reflects varying levels of injecting drug use. Findings among sex workers are consistent with studies in other regions (Chen et al. 2015; Praseeda et al. 2013; Rantala and van de Laar 2008). For prison inmates, the pooled estimates concur with the regional estimates provided by Larney et al. (2013) and are lower than those in Asia, Europe and North America.

With regard to the diagnosis of HCV infection, all studies used anti-HCV positivity as serological marker that does not distinguish between current and past HCV infection. Only a few publications evaluated HCV-RNA, and even fewer evaluated genotypes of HCV infection. This is likely related to the fact that testing for HCV-RNA and genotyping is technically more complicated and costly. Nevertheless, burden of disease for current HCV infection measured by HCV-RNA and genotyping is of great interest from an epidemiological as well as treatment response perspective.

All publications that evaluated genotypes were studies in Brazilian drug users. Data suggest that genotype 1a might not always be the predominant type (Oliveira-Filho et al. 2013). Further investigation in other countries and key populations are needed to support policies for therapeutic schemes.

Among the limitations of the study are a low sample size cutoff criteria (75 participants or greater) that were decided upon given the paucity of studies in the region and different sampling methodology that could bias prevalence results. Thirteen percent of the fifty five studies were excluded because of a small sample size, but results were not significantly different in prevalence rates from those with higher sample sizes. Another limitation is that the full data extraction was conducted by only one reviewer with an additional reviewer for only a sample of studies. While there was full agreement between reviewers for the sample reviewed by both, variations in data collection could occur for the additional studies not reviewed by both. Other limitation for providing pooled results is the high heterogeneity present among HCV prevalence in the studies. The high heterogeneity in the different analysis can be explained by differences in years of publication of the studies, inherent differences between risk groups and geographic locations. This high heterogeneity may limit the value of the regional summary measures presented; however, differences among prevalence values were expected and could be ascribed to true differences in the prevalence of infection among risk groups and geographical areas. Results can also be affected due to overlapping HIV/HCV epidemics, mainly in IDU and prison inmates. It is important to consider that HIV infection is significant in the same population groups studied for HCV and co-infection results in a more rapid progression of liver disease and HCV-related liver disease is a major cause of death for people living with HIV. Prevalence figures of HCV can be affected because the likelihood of chronic infection of HCV is higher for people living with HIV, while, on the other hand, mortality may be higher among persons co-infected by HCV and HIV.

This systematic review and meta-analysis summarized and assessed trends in prevalence of hepatitis C infection in high risk populations of the region using conventional methods for combining study data. Thus, the Forrest plots presented do not compare risk ratios or other comparative summary measures but prevalence of Hepatitis C. The search strategy included different databases such as Pubmed and Lilacs which is a specific source of scientific articles in the region; we did not include the EMBASE database due to lack of access to such database.

In conclusion, these findings emphasize the need to strengthen surveillance of hepatitis C prevalence targeted towards key populations in LAC using a broader framework of risk and vulnerability. Based on these findings, prevention and treatment strategies should include and focus on most-at-risk and vulnerable populations to respond to the epidemic of hepatitis C infection.

## Electronic supplementary material

Supplementary material 1 (PDF 1387 kb)

## Abbreviations

LAC: Latin America and the Caribbean
IDU: Injecting drug users
NIDU: Non-injecting drug users
MSM: Men who have sex with men
HCV: Hepatitis C virus
DU: Drug users
