# Supplementary material for: Hepatitis C in key populations in Latin America and the Caribbean: systematic review and meta-analysis
Source: Int J Public Health. 2015 Aug 23;60(7):789–98. doi: 10.1007/s00038-015-0708-5 (PMC4636523; doi:10.1007/s00038-015-0708-5)
Supplement: Supplementary file 1 — Supplementary material 1 (PDF 1387 kb) [file 38_2015_708_MOESM1_ESM.pdf]

Hepatitis C in Key Populations in Latin America and the Caribbean: Systematic Review and meta-analysis. International Journal of Public Health

Monica Alonso[1], Annika Gutzman[1], Rafael Mazin[1], Carlos E. Pinzon[2], Ludovic Reveiz[2], Massimo Ghidinelli[1]
[1] Pan American Health Organization, HIV/STI/TB and viral hepatitis
Washington, District of Columbia, United States
[2]Pan American Health Organization, Knowledge Management, Bioethics and Research
Washington, District of Columbia, United States
Corresponding author: Monica Alonso, Pan American Health Organization, HIV/STI/TB and viral hepatitis, 525 23rd St NW, Washington, DC 20037, United States. alonsomon@paho.org

Table 1. Overview of search terms used in the Pub Med, Lilacs and Scielo databases for the systematic review on hepatitis C in key populations in Latin America and the Caribbean

**PubMed**	**LILACS**	**SciELO**
Hepatitis OR “viral hepatitis” OR HAV OR HBV OR HCV OR HDV OR HEV		
AND<br>prevalence OR epidemiology		
AND<br>"Latin America*" OR "South America" OR "Central America" OR caribbean OR anguilla OR "Antigua and Barbuda" OR argentina OR aruba OR bahamas OR barbados OR belize OR bermuda OR Bolivia* OR Brazil* OR "British Virgin Islands" OR "Cayman Islands" OR Chile* OR Colombia* OR "Costa Rica" OR Cuba* OR dominica OR "Dominican Republic" OR "El Salvador" OR ecuador OR "French Guiana" OR grenada OR guadalupe OR guatemala OR guyana OR haiti OR honduras OR Jamaica* OR martinique OR mexico OR montserrat OR "Netherlands Antilles" OR Nicaragua* OR panama OR Paraguay* OR peru OR "Puerto Rico" OR "Saint Kitts and Nevis" OR "Saint Lucia" OR "Saint Vincent and the Grenadines" OR suriname OR "Trinidad and Tobago" OR "Turks and Caicos Islands" OR Uruguay* OR venezuela OR brasil* OR argentinean OR mexican OR costaric* OR "the Valley" OR "Saint John's" OR "Buenos Aires" OR basseterre OR basse -terre OR hamilton OR oranjestad OR nassau OR bridgetown OR belmopan OR sucre OR "La Paz" OR brasilia OR "Sao Paulo" OR "Rio de Janeiro" OR "Belo Horizonte" OR maceio OR manaus OR goiania OR belem OR "Porto Alegre" OR florianopolis OR "George Town" OR santiago OR valparaiso OR concepcion OR temuco OR bogota OR cali OR medellin OR barranquilla OR "San Jose" OR havana OR habana OR "Santo Domingo" OR roseau OR "San Salvador" OR quito OR guayaquil OR "Saint George's" OR "Port au Prince" OR "Port of Spain" OR tegucigalpa OR kingston OR kingston OR willemstad OR managua OR asuncion OR lima OR "San Juan" OR marigo OR castres OR paramaribo OR montevideo OR caracas OR "Road Town" OR menicagli OR "Tuxtla Gutierrez" OR chihuahua OR guanajuato OR guadalajara OR toluca OR morelia OR monterrey OR puebla		

Table 2. Baseline characteristics of included studies for prevalence of current or past Hepatitis C infection in Latin America and the Caribbean from 2000 to 2013

Population	Geographical area	Subpopulation	Pos/ Total	Field Work	Author	Prevalence%	high CI95%	low CI 95%
Female Sex Workers	Argentina (Buenos Aires)		12/ 274	2000- 2002	Pando 2006	4.40	7.50	2.52
	Argentina (Córdoba)		2/ 86	2000- 2002	Pando 2006	2.30	8.09	0.64
	Argentina (La Plata)		2/ 100	2000- 2002	Pando 2006	2.00	7.00	0.55
	Argentina (Salta)		8/ 98	2000- 2002	Pando 2006	8.20	15.28	4.19
	Brazil (Tubarão, Laguna, Imbituba)	91,2% female	13/ 147	2009	Schuelter- Trevisol 2013	8.80	14.54	5.24
	Panama (Bocas del Torro)		1/ 95	2009- 2011	Hakre 2013	1.10	5.72	0.19
	Panama (Chiriqui)		0/ 155	2009- 2011	Hakre 2013	0.00	2.42	0.00
	Panama (Ciudad de Panama)		1/ 455	2009- 2011	Hakre 2013	0.20	1.23	0.04
	Panama (Colón)		0/ 150	2009- 2011	Hakre 2013	0.00	2.50	0.00
	Peru (Iquitos)		4/ 200	2003- 2004	Amaya 2007	2.00	5.03	0.78
	Peru (Lima)		0/ 98	≤ 2003	Valdivia 2003	0.00	3.77	0.00
	Venezuela (Los Teques)		1/ 212	1999	Camejo 2003	0.50	2.62	0.08
Trans SW	Argentina (7 cities)		12/ 264	2006- 2009	Dos Ramos Farías 2011	4.50	7.78	2.62
	Uruguay (Montevideo)		13/ 200	1999	Russi 2003	6.50	10.80	3.84
Male SW	Argentina (7 cities)		5/ 82	2006- 2009	Dos Ramos Farías 2011	6.10	13.50	2.63
MSM	Argentina (Buenos Aires)		37/ 496	2007- 2009	Pando 2012	7.50	10.11	5.46
			7/ 877	2003	Segura 2010	0.80	1.64	0.39
			13/ 681	2000- 2001	Pando 2006	1.90	3.24	1.12
	Dominican Republic (Barahona)	MSM+ Trans	19/ 281	≤ 2013	Johnston 2013	6.90	10.32	4.37
	Dominican Republic (La Altagracia)	MSM+ Trans	14/ 270	≤ 2013	Johnston 2013	5.00	8.52	3.12
	Dominican Republic (Santiago)	MSM+ Trans	7/ 327	≤ 2013	Johnston 2013	2.00	4.35	1.04
	Dominican Republic (Santo Domingo)	MSM+ Trans	4/ 510	≤ 2013	Johnston 2013	0.80	1.99	0.30
Prisoners	Brazil (Espírito Santo)	Female	23/ 121	1997	Miranda 2000	19.00	26.91	13.01
	Brazil (Goiás)	Female	9/ 148	2007- 2008	Barros 2013	6.10	11.15	3.23
		Male	40/ 270	2004	Gonçalves 2005	14.80	19.54	11.07
		Male DU	38/ 185	2004	Gonçalves 2005	20.54	26.93	15.35
	Brazil (Mato Grosso do Sul)		33/ 686	2009	Pompilio 2011	4.80	6.68	3.45
	Brazil (Ribeirão Preto)	Male	29/ 333	2003	Coelho 2009	8.70	12.23	6.13
	Brazil (Rio Grande do Sul)	Female	13/ 76	2006	Gabe 2008	17.12	27.10	10.28
	Brazil (Santa Cruz do Sul)		19/ 195	2010- 2011	Da Rosa 2012	9.70	14.71	6.32
	Brazil (São Paulo)	Female	47/ 290	2000	Strazza 2004	16.20	20.89	12.41
		Male	310/ 756	1993- 1994	Guimarães 2001	41.00	44.55	37.56
	Brazil (São Vincente)	Male	27/ 514	2007	Maerrawi 2012	5.30	7.53	3.63
	Brazil (Sergipe)		13/ 422	2009- 2010	Santos 2011	3.10	5.20	1.81
	Mexico (Durango)		19/ 181	2001- 2002	Alvarado- Esquivel 2005	10.00	15.82	6.83
	Venezuela (Maracaibo)	Male	3/ 200	≤ 2009	Monsalve- Castillo 2009	1.50	4.32	0.51
DU	Argentina (Buenos Aires)	past/present cocaine/coca paste	61/ 203	2005- 2006	Sheehan 2012	30.00	36.68	24.16
	Argentina(unk, Buenos Aires)	rehab center; 43% ever-IDU	68/ 101	≤ 2003	Cocozella 2003	67.30	75.70	57.70
	Brazil (26 sites)	Mental health patients	32/ 529	2005- 2007	Carmo 2013	6.10	8.42	4.32
	Brazil (Cuiabá)	Male, past/present DU enroll tre..	20/ 314	2006- 2007	Novais 2009	6.40	9.63	4.16
	Brazil (Goiânia, Campo Grande)	enrollment in drug treatment ce..	48/ 691	2005- 2006	Lopes 2009	6.90	9.09	5.28
	Brazil (Pará)	cocaine users	124/ 384	2010- 2011	Oliveira-Filho 2013	32.30	37.12	27.81
	Brazil (Rio de Janeiro)		13/ 225	≤ 2000	Bastos 2000	5.80	9.64	3.41
	Brazil (São José dos Pinhais)		8/ 589	≤ 2012	Rodrigues Neto 2012	1.36	2.66	0.69
	Columbia (Bucaramanga)	enroll drug treatm. center, >3 m..	0/ 90	2009	Amorocho 2011	0.00	4.09	0.00
		Prisoners	0/ 169	2009	Amorocho 2011	0.00	2.22	0.00
	Peru (Lima)	enrollment in drug treatment ce..	4/ 203	1999	Sánchez 2000	2.00	4.96	0.77
	Venezuela (Maracaibo)	enrollment in drug treatment ce..	2/ 100	2004-2005	Monsalve- Castillo 2007	2.00	7.00	0.55
IDU	Argentina (Buenos Aires)		95/ 174	2000- 2001	Weissenbacher 2003	54.60	61.82	47.18
	Brazil (5 cities)		150/ 287	1998	Caiaffa 2006	52.30	57.97	46.49
	Brazil (6 cities)		393/ 857	2000-2001	Caiaffa 2006	45.80	49.21	42.55
	Brazil (Goiânia, Campo Grande)		32/ 102	2005- 2006	Lopes 2009	31.40	40.91	23.18
	Brazil (Pará)	injected at least once in lifetime	37/80	2010- 2011	Oliveira-Filho 2013	46.25	57.10	35.80
	Brazil (Rio de Janeiro)		102/ 606	1999- 2001	Oliveira 2006	16.80	20.02	14.06
	Columbia (Bogotá)		5/ 296	≤ 2004	Mejia 2004	1.70	3.89	0.72
	Mexico (Ciudad Juárez)		193/ 202	2005	Frost 2006	95.50	97.64	91.74
	Mexico (Tijuana)		212/ 222	2005	Frost 2006	95.50	97.54	91.91
	Puerto Rico (San Juan)		331/ 372	≤ 2006	Reyes 2006	89.00	91.77	85.39
			30/ 77	2001-2002	Pérez 2005	39.60	50.13	28.84
	Uruguay (Montevideo)		43/ 200	2003	Osimani 2005	21.50	27.70	16.37
NIDU	Argentina (Buenos Aires)		8/ 146	2006-2007	Muzzio 2010	5.50	10.44	2.80
			38/ 504	2002- 2003	Rossi 2008	7.50	10.18	5.54
	Brazil (all macro regions)	Inhaling	9/ 265	2005-2009	Pereira 2013	3.40	6.33	1.80
		Smoking	38/ 1839	2005-2009	Pereira 2013	2.10	2.83	1.51
		Sniffing	25/ 749	2005-2009	Pereira 2013	3.30	4.88	2.27
	Brazil (Cuiabá)		4/ 266	2006- 2007	Novais 2009	1.50	3.80	0.58
	Brazil (Goiânia, Campo Grande)		16/ 589	2005- 2006	Lopes 2009	2.70	4.37	1.68
	Brazil (Goiania, Campo Grande, Cuiabá)		20/ 852	2005- 2006	Ferreira 2009	2.35	3.60	1.53
	Brazil (Pará)		83/ 304	2010- 2011	Oliveira-Filho 2013	27.30	32.57	22.60
	Brazil (Rio de Janeiro)		0/ 81	2010- 2011	Santos Cruz 2013	0.00	4.53	0.00
			5/ 201	≤ 2000	Bastos 2000	2.50	5.69	1.07
	Brazil (Rio Grande do Sul)		10/ 119	2007	Germano 2010	8.40	14.78	4.63
	Brazil (Salvador)		1/ 79	2010- 2011	Santos Cruz 2013	1.30	6.83	0.23
			3/ 125	2001- 2002	Nunes 2007	2.40	6.82	0.82
	Mexico (Jalisco, Colima, Michoacán)		5/ 122	2007	Campollo 2012	4.10	9.24	1.76
	Uruguay (Montevideo)		37/ 267	2002- 2003	Osimani 2003	10.10	18.52	10.23

Table 2. continued. Baseline characteristics of included studies for current Hepatitis C infection in Latin America and the Caribbean from 2000 to 2013

Population	Geographical area	Subpopulation	Pos/ Total	Field work	Author	Prevalence %	higher CI 95%	lower CI 95%
Prisoners	Brazil (Goiás)	Female	5/ 148	2007- 2008	Barros 2013	0.03	7.67	1.45
	Brazil (Mato Grosso do Sul)		20/ 686	2009	Pompilio 2011	0.03	4.47	1.90
	Brazil (Sergipe)		11/ 422	2009- 2010	Santos 2011	0.03	4.61	1.46
	Mexico (Durango)		16/ 181	2001- 2002	Alvarado- Esquivel 2005	0.09	13.88	5.51
	Venezuela (Maracaibo)	Male	3/ 200	≤ 2009	Monsalve- Castillo 2009	0.02	4.32	0.51
DU	Argentina (unknown)		41/ 101	≤ 2003	Cocozella 2003	0.41	50.34	31.53
	Brazil (Cuiabá)	Male	16/ 314	2006- 2007	Novais 2009	0.05	8.12	3.16
	Brazil (Pará)		120/ 384	2010- 2011	Oliveira-Filho 2013	0.31	36.05	26.82
	Venezuela (Maracaibo)		1/ 100	2004-2005	Monsalve- Castillo 2007	0.01	5.45	0.18
IDU	Brazil (Para)		37/ 80	2010- 2011	Oliveira-Filho 2013	0.46	57.10	35.75
NIDU	Brazil (Para)		83/ 304	2010- 2011	Oliveira-Filho 2013	0.27	32.57	22.60

Notes: DU: drug users; IDU: injecting drug users; NIDU: non injecting drug users; MSM : men who have sex with men; SW: sex workers.

Table 3. Quality assessment of included studies review on prevalence of current or past Hepatitis C infection in Latin America and the Caribbean from 2000 to 2013

Year published	Author	Country	Sampling method	Measure outcome variable	Response rate	Adequacy Sample Size	Privacy of participants to respond	Definition of Key population	Definition of key outcome
2000	Bastos	Brazil	Non probabilistic	Laboratory test	>60%	sample >100	Yes	Yes	Laboratory
	Miranda	Brazil	Non probabilistic	Laboratory test	>60%	sample >100	Yes	Yes	Laboratory
	Sanchez	Peru	Unknown	Laboratory test	unknown	sample >100	Yes	Yes	Laboratory
2001	Guimaraes	Brazil	Unknown	Laboratory test	unknown	sample >100	Yes	Yes	Laboratory
2003	Camejo	Venezuela	Unknown	Laboratory test	unknown	sample >100	No	No	Laboratory
	Cocozella	Argentina	Non probabilistic	Laboratory test	unknown	sample >100	No	Yes	Laboratory
	Osimani	Uruguay	Non probabilistic	Laboratory test	unknown	sample >100	Yes	Yes	Laboratory
	Russi	Uruguay	Non probabilistic	Laboratory test	>60%	sample >100	Yes	No	Laboratory
	Valdivia	Peru	Unknown	Laboratory test	unknown	75-100 participants	No	No	Laboratory
	Weissenbacher	Argentina	Non probabilistic	Laboratory test	unknown	sample >100	Yes	Yes	Laboratory
2004	Mejia	Columbia	Non probabilistic	Laboratory test	unknown	sample >100	No	No	Laboratory
	Strazza	Brazil	Unknown	Laboratory test	>60%	sample >100	Yes	Yes	Laboratory
2005	Alvarado- Esquivel	Mexico	Probabilistic	Laboratory test	>60%	sample >100	Yes	Yes	Laboratory
	Goncalves	Brazil	Non probabilistic	Laboratory test	unknown	sample >100	Yes	Yes	Laboratory
	Osimani	Uruguay	Non probabilistic	Laboratory test	unknown	sample >100	Yes	Yes	Laboratory
	Perez	Puerto Rico	Probabilistic	Laboratory test	>60%	75-100 participants	Yes	Yes	Laboratory
2006	Caiaffa	Brazil	Non probabilistic	Laboratory test	unknown	sample >100	Yes	Yes	Laboratory
	Frost	Mexico	Probabilistic	Laboratory test	>60%	sample >100	Yes	Yes	Laboratory
	Oliveira	Brazil	Non probabilistic	Laboratory test	unknown	sample >100	Yes	Yes	Laboratory
	Pando	Argentina	Non probabilistic	Laboratory test	>60%	75-100 participants	Yes	Yes	Laboratory
						sample >100	Yes	Yes	Laboratory
	Reyes	Puerto Rico	Unknown	Laboratory test	unknown	sample >100	Unkown	Yes	Laboratory
2007	Guerra Amaya	Peru	Non probabilistic	Laboratory test	unknown	sample >100	Unkown	No	Laboratory
	Monsalve- Castillo	Venezuela	Unknown	Laboratory test	unknown	75-100 participants	Unkown	Yes	Laboratory
	Nunes	Brazil	Non probabilistic	Laboratory test	>60%	sample >100	Yes	Yes	Laboratory
2008	Gabe	Brazil	Unknown	Laboratory test	unknown	75-100 participants	Unkown	Yes	Laboratory
	Rossi	Argentina	Non probabilistic	Laboratory test	unknown	sample >100	Yes	Yes	Laboratory
2009	Coelho	Brazil	Probabilistic	Laboratory test	>60%	sample >100	Unkown	Yes	Laboratory
	Ferreira	Brazil	Non probabilistic	Laboratory test	unknown	sample >100	Yes	Yes	Laboratory
	Lopes	Brazil	Non probabilistic	Laboratory test	>60%	sample >100	Yes	Yes	Laboratory
	Monsalve- Castillo	Venezuela	Probabilistic	Laboratory test	unknown	sample >100	Unkown	Yes	Laboratory
	Novais	Brazil	Non probabilistic	Laboratory test	>60%	sample >100	Yes	Yes	Laboratory
2010	Germano	Brazil	Non probabilistic	Laboratory test	unknown	sample >100	Unkown	No	Laboratory
	Muzzio	Argentina	Non probabilistic	Laboratory test	unknown	sample >100	Yes	Yes	Laboratory
	Segura	Argentina	Non probabilistic	Laboratory test	unknown	sample >100	Unkown	Yes	Laboratory
2011	Bautista Amorocho	Columbia	Non probabilistic	Laboratory test	unknown	75-100 participants	Unkown	Yes	Laboratory
						sample >100	Unkown	Yes	Laboratory
	Campollo	Mexico	Non probabilistic	Laboratory test	>60%	sample >100	Unkown	Yes	Laboratory
	Dos Ramos Farias	Argentina	Non probabilistic	Laboratory test	unknown	75-100 participants	Yes	Yes	Laboratory
						sample >100	Yes	Yes	Laboratory
	Pompilio	Brazil	Non probabilistic	Laboratory test	unknown	sample >100	Unkown	Yes	Laboratory
	Santos	Brazil	Non probabilistic	Laboratory test	>60%	sample >100	Yes	Yes	Laboratory
	Sheehan	Argentina	Unknown	Laboratory test	unknown	sample >100	Unkown	Yes	Laboratory
2012	Carmo	Brazil	Probabilistic	Laboratory test	unknown	sample >100	Yes	Yes	Laboratory
	Maerrawi	Brazil	Non probabilistic	Laboratory test	>60%	sample >100	Yes	Yes	Laboratory
	Pando	Argentina	Probabilistic	Laboratory test	unknown	sample >100	Unkown	Yes	Laboratory
	Rodrigues	Brazil	Non probabilistic	Laboratory test	unknown	sample >100	Yes	No	Laboratory
	Rosa	Brazil	Probabilistic	Laboratory test	unknown	sample >100	Unkown	Yes	Laboratory
2013	Barros	Brazil	Non probabilistic	Laboratory test	>60%	sample >100	Yes	Yes	Laboratory
	Hakre	Panama	Probabilistic	Laboratory test	>60%	75-100 participants	Yes	Yes	Laboratory
						sample >100	Yes	Yes	Laboratory
	Johnston	Dominican Republic	Probabilistic	Laboratory test	unknown	sample >100	Yes	Yes	Laboratory
	Oliverira-Filho	Brazil	Non probabilistic	Laboratory test	unknown	75-100 participants	Unkown	Yes	Laboratory
						sample >100	Unkown	Yes	Laboratory
	Pereira	Brazil	Probabilistic	Laboratory test	>60%	sample >100	Yes	Yes	Laboratory
	Santos Cruz	Brazil	Non probabilistic	Laboratory test	unknown	75-100 participants	Yes	Yes	Laboratory
	Schuelter- Trevisol	Brazil	Non probabilistic	Laboratory test	unknown	sample >100	Unkown	No	Laboratory
